# Total 25-Hydroxyvitamin D Determination by an Entry Level Triple Quadrupole Instrument: Comparison between Two Commercial Kits

**DOI:** 10.1155/2013/270426

**Published:** 2013-02-28

**Authors:** Jacopo Gervasoni, Andrea Cocci, Cecilia Zuppi, Silvia Persichilli

**Affiliations:** Istituto di Biochimica e Biochimica Clinica, Università Cattolica del Sacro Cuore, Largo A. Gemelli 8, 00168 Rome, Italy

## Abstract

*Objective*. 25-hydroxyvitamin D_2_/D_3_ (25-OHD_2_/D_3_) determination is a reliable biomarker for vitamin D status. Liquid chromatography-tandem mass spectrometry was recently proposed as a reference method for vitamin D status evaluation. The aim of this work is to compare two commercial kits (Chromsystems and PerkinElmer) for 25-OHD_2_/D_3_ determination by our entry level LC-MS/MS. *Design and Methods*. Chromsystems kit adds an online trap column to an HPLC column and provides atmospheric pressure chemical ionization, isotopically labeled internal standard, and 4 calibrator points. PerkinElmer kit uses a solvent extraction and protein precipitation method. This kit can be used with or without derivatization with, respectively, electrospray and atmospheric pressure chemical ionization. For each analyte, there are isotopically labeled internal standards and 7 deuterated calibrator points. *Results*. Performance characteristics are acceptable for both methods. Mean bias between methods calculated on 70 samples was 1.9 ng/mL. Linear regression analysis gave an *R* ^2^ of 0.94. 25-OHD_2_ is detectable only with PerkinElmer kit in derivatized assay option. *Conclusion*. Both methods are suitable for routine. Chromsystems kit minimizes manual sample preparation, requiring only protein precipitation, but, with our system, 25-OHD_2_ is not detectable. PerkinElmer kit without derivatization does not guarantee acceptable performance with our LC-MS/MS system, as sample is not purified online. Derivatization provides sufficient sensitivity for 25-OHD_2_ detection.

## 1. Introduction

Vitamin D (vitD) is is critical for the regulation of calcium and phosphate homeostasis and is implicated in important biological processes [1]. VitD exists in two forms; vitD_3_ (cholecalciferol) is formed in the skin upon exposure to sunlight; vitD_2_ (ergocalciferol) is obtained from the ultraviolet irradiation of plants materials. These two vitD forms are metabolized in the liver to give 25-hydroxy vitamin D (25-OHD), further hydrolyzed in the kidney to biologically active metabolite 1,25-dihydroxy vitamin D (1,25-OHD). This last metabolite is difficult to measure, because it is present at extremely low concentrations (15–60 pg/mL), thus, circulating liver metabolites 25-OHD_2_/D_3_ are recognized as markers for vitamin D status [2, 3].

Recently vitD status has been associated with several diseases including cancer cardiovascular disease, diabetes, multiple sclerosis, osteoporosis, rheumatoid arthritis, and chronic pain [4]. 

Methods for 25-OHD_3_ and 25-OHD_2_ determination can be grouped in immunochemical methods (based on radioactive, enzymatic, or chemiluminescence detection) and chromatographic methods (HPLC and LC-MS/MS). 

Immunoassay methods are the most used for their rapidity, despite that several studies reported problems of reproducibility and interferences. Recent papers have reviewed performance and limitations of immunochemical, HPLC-based, and liquid chromatography-tandem mass spectrometry (LC-MS/MS) methods [5, 6]. One limitation in the 25-OHD_2_/D_3_ measurement was represented by the lack of reference standards until the development of two ethanol-based calibrators (SRM 2972) and four serum-based calibrators (SRM 972) by the US National Institute of Standards and Technology (NIST). There are two LC-MS/MS methods as reference methods by the Joint Committee for Traceability in Laboratory Medicine [7, 8].

The release of these standards significantly contributed to increasing the agreement among different LC-MS/MS methods, overcoming the lack of standardization highlighted by the Vitamin D External Quality Assessment Schemes (DEQAS) [9].

The purpose of this work is to compare two commercial kits for 25-OHD_2_/D_3_ determination by LC-MS/MS with our entry level triple quadrupole instrument.

## 2. Materials and Methods

Serum samples were obtained from 70 subjects belonging to the hospital staff (40 males, 30 females; 18–60 age range) and submitted to periodical clinical control. All subjects have given their informed consent. Samples were collected and stored at −80°C. When thawed, all samples were analyzed for 25-OHD_2_/D_3_ by LC-MS/MS with both Chromsystems (Chromsystems Instruments & Chemicals GmbH, Munchen, Germany) and PerkinElmer (Wallac Oy, Turku, Finland) kits. 

Water, acetonitrile, and formic acid (LC-MS grade) were purchased from Baker (Mallinckrodt Baker Italy, Milan, Italy).

Analyses were performed using a triple stage quadrupole TSQ Quantum Access (Thermo Fisher, Palo Alto, CA, USA) equipped with APCI source for Chromsystems and for PerkinElmer in nonderivatized mode and with ESI source for PerkinElmer in derivatized mode.

### 2.1. Chromsystems Assay

In the Chromsystems kit, the preanalytical phase is simplified to only a protein precipitation, because an online trap column concentrates the analytes and separates interfering substances. The trap column is connected to an HPLC column which provides further purification. 

Calibration was performed using a lyophilized multilevel serum calibrator set NIST traceable (*n* = 6) of known concentration. Low and high concentration lyophilized sera samples were used as quality controls. ^2^H_6_-25(OH)D_3_ was used as internal standard (IS) to correct for sample treatment and instrument variability.

Samples, calibrators, and quality controls were prepared according to the manufacturer's instructions. Briefly, to 100 *μ*L of serum sample, 25 *μ*L of precipitation reagent and 200 *μ*L of internal standard were added. After an incubation of 10 minutes, samples were centrifuged at 15000 ×g for 5 minutes, and 200 *μ*L was transferred into the vials. 50 *μ*L was injected into the HPLC-MS/MS system.

Samples were analyzed using an APCI source to maximize sensitivity. The APCI source was working in the positive mode, producing positively charged ions in the form of [H^+^] adduct ions. Discharge current was maintained at 7.0 *μ*A; vaporizer temperature was maintained at 400°C with a capillary temperature of 300°C. Gas settings were sheath gas pressure, 40 (arbitrary units); auxiliary gas pressure, 35 (arbitrary units); ion sweep gas pressure, 0 (arbitrary units). Argon was used as collision gas at a collision pressure of 1.5 mTorr. 

Mobile phases (A and B) were used independently in isocratic mode with trap column, and analytical column respectively. All materials and reagents were provided by the manufacturers. Total run time was 6.5 minutes.

The selected reaction monitoring (SRM) transitions for each analyte, their respective collision energy, and tube lens values were reported in Table 1(a).

### 2.2. PerkinElmer Assay

PerkinElmer kit uses a combination of solvent extraction and protein precipitation procedures. This kit can be alternatively used with derivatization, using ESI source, or without derivatization, using APCI. Derivatization improves ionization efficiency and MS/MS signal intensity of the analytes. In our preliminary tests, our entry-level mass spectrometer showed an inadequate sensitivity for quantification of serum 25(OH)D_2_ and 25(OH)D_3_ using the kit in nonderivatized mode (data not shown). Kit was then used only in derivatized mode. Calibration was performed using 7 calibrator points (charcoal stripped human serum enriched with six increasing levels of ^2^H_6_-25(OH)D_2_ and ^2^H_6_-25(OH)D_3_). Three control levels (lyophilized serum added with increasing amount of ^2^H_6_-25(OH)D_2_ and ^2^H_6_-25(OH)D_3_) were used as quality controls.

For each analyte specific isotopically labeled internal standards (^2^H_3_-25(OH)D_2_ for VitD_2_ and ^2^H_3_-25(OH)D_3_ for VitD_3_) were used.

Samples, calibrators, and quality controls were prepared according to the manufacturer's instructions with slightly modifications in order to enhance sensitivity. Briefly, to 150 *μ*L of serum sample, 300 *μ*L of daily precipitation solution (DPS, IS solution in acetonitrile 0.1% formic acid) was added. After an incubation of 10 minutes samples were centrifuged at 15000 ×g for 5 minutes, and 300 *μ*L was transferred into 96 well plates. Samples were placed under a stream of high purity dry nitrogen gas until all the samples were dry. 50 *μ*L of derivatization reagent was added to each wel,l and the plate, covered with aluminum foil, was shaken at 750 rpm for 10 minutes. After removing the aluminum foil 50 *μ*L of quench solution was added to each well, and the plate, covered with aluminum foil, was shaken at 750 rpm for 10 minutes. The plate was loaded onto the autosampler, and 50 *μ*L were injected into the HPLC-MS/MS system.

For derivatizated assay, among the columns proposed, we selected a Waters XBridge C8 (3.5 *μ*m 2.1 × 100 mm) equilibrated with a gradient of water/methanol added with 0.1% formic acid and 0.025% additive (provided by the manufacturer). While, for nonderivatized assay, we used a C18 Beta Basic 5 *μ*m 2.1 × 100 mm column equilibrated with a different gradient of water/methanol added with 0.1% formic acid. Total run time was 9.0 and 6.0 minutes for derivatized and underivatized assays, respectively.

In derivatized assay, the ESI source was working in the positive mode, producing positively charged ions in the form of [H^+^] adduct ions. Capillary voltage was maintained at 3800 V, with a capillary temperature of 300°C. Gas settings were sheath gas pressure, 40 (arbitrary units); auxiliary gas pressure, 5 (arbitrary units); ion sweep gas pressure, 0 (arbitrary units). Argon was used as collision gas at a collision pressure of 1.5 mTorr.

The MRM transitions for each analyte, their respective collision energy, and tube lens values were reported in Table 1(b).

In non-derivatizated assay, the APCI source was working in positive mode, producing positively charged ions in the form of [H^+^] adduct ions. Discharge current was maintained at 7.0 *μ*A; vaporizer temperature was maintained at 430°C with a capillary temperature of 300°C. Gas settings were sheath gas pressure, 40 (arbitrary units); auxiliary gas pressure, 5 (arbitrary units); ion sweep gas pressure, 0 (arbitrary units). Argon was used as collision gas at a collision pressure of 1.5 mTorr.

### 2.3. Methods Evaluation

The following parameters were assessed: linearity, limit of quantification (LOQ), limit of detection (LOD), and imprecision.

LOD and LOQ were evaluated by measuring the lower calibration point serially diluted with water.

The imprecision was evaluated using all the quality controls (QCs), three for PerkinElmer and two for Chromsystems. To evaluate within-assay imprecision, each QC was measured ten times in the same analytical run; between-assay imprecision was evaluated by measuring in duplicate the same QC samples for ten consecutive days.

25-OHD_3_ values obtained by the LC-MS/MS methods were correlated using linear regression analysis. The bias of results was analyzed according to Bland-Altman [10].

Data acquisition was carried out using the mass spectrometer software (Excalibur 2.0.7, Thermo Fisher, Palo Alto, CA, USA). Quantitative analyses were carried out using Excalibur software for the Chromsystems method and Microsoft Excel 2010 (Microsoft Office 2010) for the PerkinElmer method. Statistical analysis was performed using Microsoft Excel 2010 (Microsoft Office 2010).

## 3. Results

**Figure 1:**
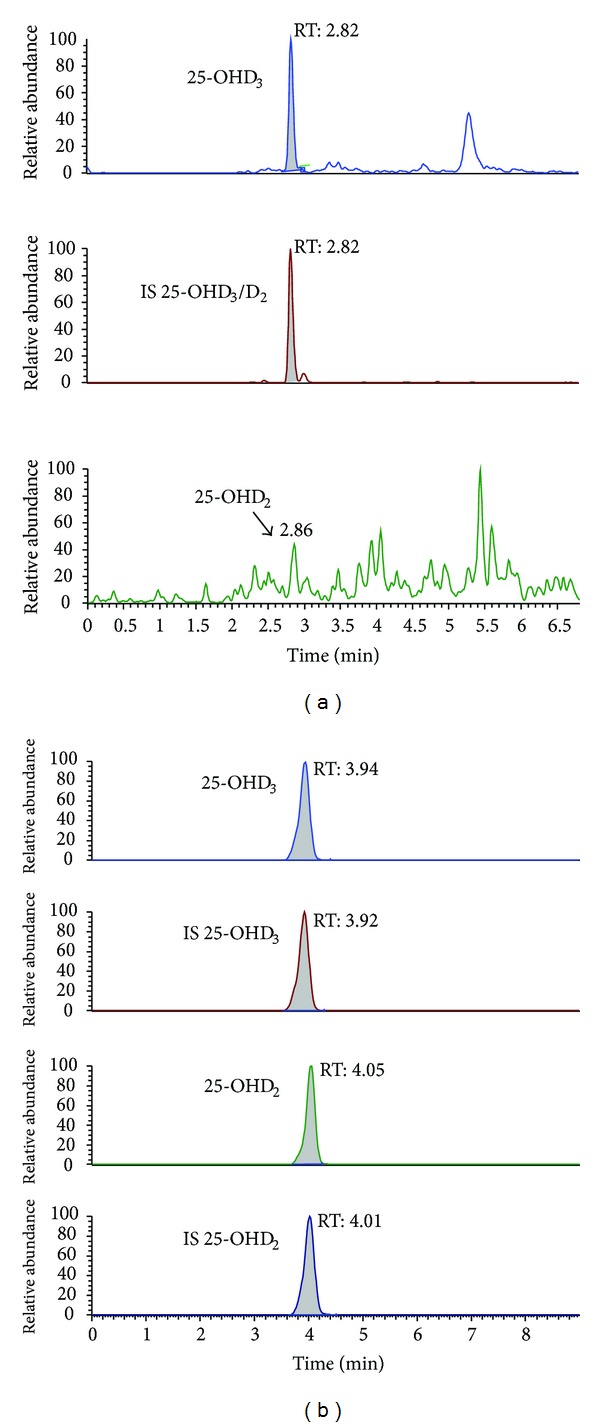
Typical SRM chromatograms of a serum sample (23.4 ng/mL 25-OHD_3_ and 25-OHD_2_ not detectable) assayed using the Chromsystems kit. The retention time of 25-OHD_2_/D_3_ is 2.8 minutes (a). Typical SRM chromatogram of a serum sample (21.8 ng/mL 25-OHD_3_ and 25-OHD_2_ 1.6 ng/mL) assayed using the PerkinElmer kit. The retention time is 4.0 min (b).

Figure 1(a) shows, from top to bottom, typical SRM chromatograms of 25-OHD_3_, IS (^2^H_6_-25(OH)D_3_), and 25-OHD_2_ obtained using the Chromsystems kit. The retention time of these analytes is approximately 2.8 min.

Figure 1(b) shows, from top to bottom, typical SRM chromatogram of 25-OHD_3_, IS ^2^H_3_-25(OH)D_3_, 25-OHD_2_ and IS ^2^H_3_-25(OH)D_2_ obtained using the PerkinElmer kit. The retention time is approximately 4.0 min.

In derivatized assay option, chemical modification of the 25-OHD_2_ and 25-OHD_3_ is achieved by derivatizing them in the extracted serum using 4-Phenyl-1,2,4-triazoline-3,5-dione (PTAD). In this reaction, PTAD is selectively added to the cis-diene double bonds of the 25-OHD_2_/D_3_ molecules, resulting in the generation of a new chiral center and the subsequent formation of a new pair of 25-OHD_2_/D_3_ diastereoisomers. Although partially resolved both 6S- and 6R-isomers signals should be finally combined for quantitative determination of 25-OHD_2_ and 25-OHD_3_ in the samples. This could explain the poor peak shape showed in Figure 1(b).

Both chromatographic runs are without interferences, confirming the high selectivity of these methods.

Performance characteristics are acceptable for both methods. According to manufacturer's instructions the linearity of the PerkinElmer assay is 329 ng/mL for 25-OHD_2_ and 314 ng/mL for 25-OHD_3_, and the linearity of the Chromsystems assay is 250 ng/mL for both 25-OHD_2_ and 25-OHD_3_.

LOQ, estimated as the lowest concentration of 25-OHD_3_ where the relative uncertainty of a single measurement is reproducible within ±20%, was 3.0 ng/mL for PerkinElmer kit and 4.1 ng/mL for Chromsystems kit. 

LOD, defined as the minimum concentration of 25-OHD_3_ which gives a signal three times higher than the noise, was 1.6 ng/mL for PerkinElmer kit and 2.6 ng/mL for Chromsystems kit.

LOQ for 25-OHD_2_ was 1.4 ng/mL for PerkinElmer kit and 3.0 for Chromsystems kit; the LOD of 25-OHD_2_ was 0.5 ng/mL for PerkinElmer kit and 2.3 ng/mL for Chromsystems kit. Since 25-OHD_2_ is normally present in very low concentrations (lower than 2.0 ng/mL), its determination is not possible with our entry level instrument using Chromsystems kit. Moreover, also using a slightly more performing instrument, as reported in the package insert of the Chromsystems kit, LOQ (2.4 ng/mL) is not adequate for the quantification of 25-OHD. 

Intra-assay imprecision for 25-OHD_3_ ranged from 3.6% to 3.7% for Chromsystems and from 4.6% to 4.9% for PerkinElmer. Interassay imprecision for 25-OHD_3_ ranged from 4.6% to 4.8% for Chromsystems and from 4.2% to 5.1% for PerkinElmer.

Intra-assay imprecision for 25-OHD_2_ ranged from 3.8% to 4.0% for Chromsystems and from 4.5% to 4.8% for PerkinElmer. Interassay imprecision for 25-OHD_2_ ranged from 5.7% to 6.6% for Chromsystems and from 4.3% to 5.1% for PerkinElmer.

**Figure 2:**
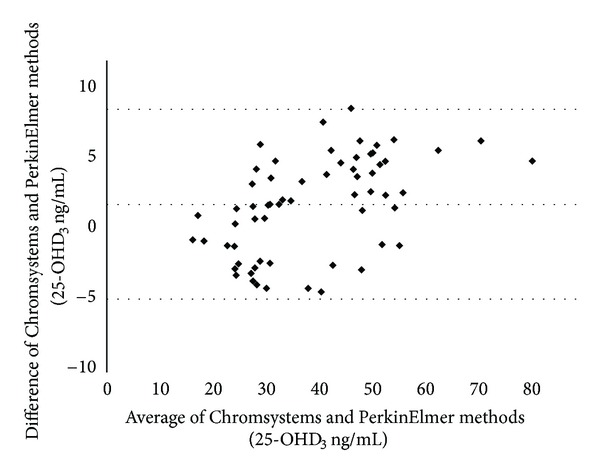
Bland-Altman plots of the differences between 25-OHD_3_ values obtained by Chromsystems and PerkinElmer kits. Mean ± 1.96 SD.

Figure 2 shows the Bland-Altman plots of the differences between 25-OHD_3_ values obtained with Chromsystems and PerkinElmer methods. Mean bias was 1.9 ng/mL, showing a good agreement between the two methods, confirmed by linear regression analysis (*R* ^2^: 0.94; *Y*_Chromsystems_ = 1.15*X*_PerkinElmer_ − 8.44). 

## 4. Conclusion

Using the Chromsystems kit, manual sample preparation is minimized and limited to a simple and effective protein precipitation. 

The PerkinElmer kit without derivatization does not guarantee acceptable performance with our LC-MS/MS system, but the manufacturer reports better analytical performance using a more performant instrument. Derivatization is more time consuming but provides sufficient sensitivity for the detection of 25-OHD_2_.

Chromsystems kit does not declare neither the type of the column nor the composition of mobile phases, while PerkinElmer does.

In PerkinElmer kit, automated calculation is made highly complicated by the presence of different deuterated standards and cannot be performed by our software.

Several studies indicate the presence of 25-OHD_2_/D_3_ epimers, particularly 3 epi-25OH-D_2_ and 3 epi-25OH-D_3_, like potential confounders in 25-OHD_2_/D_3_ measurements. The presence of these epimers was initially considered relevant only for children younger than one year [11], but recently work showed that the concentration of 25-OHD_2_/D_3_ epimers may also be significant in adults [12].

For these reasons, both PerkinElmer and Chromsystems provide an alternative kit that permits to discriminate and quantify 25-OHD_2_/D_3_ epimers.

Therefore as a future perspective, we intend to clarify the possible role of epimers in adults using LC-MS/MS methods able to separate the less biologically active 25-OHD_2_/D_3_ epimers.

## Figures and Tables

**Table 1a:** (a)

Chromsystems			
Compound	SRM	Collision Energy [eV]	Tube Lens [V]
25(OH)D_3_	383.2 → 210.8	23	80
25(OH)D_2_	395.2 → 268.8	17	80
25(OH)D_3_/D_2_ IS	389.3 → 210.8	28	80

**Table 1b:** (b)

PerkinElmer			
Compound	SRM	Collision Energy [eV]	Tube Lens [V]
25(OH)D_3_ Cal	613.4 → 298.1	19	110
25(OH)D_3_ IS	610.1 → 310.1	19	110
25(OH)D_3_	607.4 → 298.1	19	110
25(OH)D_2_ Cal	625.4 → 298.1	17	110
25(OH)D_2_ IS	622.4 → 301.1	17	110
25(OH)D_2_	619.4 → 298.1	17	110
